# Generative diffusion models for spatiotemporal influenza forecasting

**Published:** 2026-04-27

**Authors:** Joseph Lemaitre, Justin Lessler

**Affiliations:** 1 Department of Epidemiology, Gillings School of Global Public Health, University of North Carolina at Chapel Hill, Chapel Hill, NC 27599, USA; 2 Department of Epidemiology, Johns Hopkins Bloomberg School of Public Health, Baltimore, MD 21205, USA; 3 Carolina Population Center, University of North Carolina at Chapel Hill, Chapel Hill, NC 27599, USA

## Abstract

Forecasting infectious disease incidence can provide important information to guide public health planning, yet is difficult because epidemic dynamics are complex. Current mechanistic and statistical approaches often struggle to capture multimodal uncertainty or emergent trends. Influpaint adapts denoising diffusion probabilistic models to epidemic forecasting. By encoding influenza seasons as spatiotemporal images in which pixel intensity represents incidence, Influpaint learns a rich distribution of disease dynamics from a hybrid dataset of surveillance and simulated trajectories. Forecasting is formulated as a conditional generation (inpainting) task from partial observations. We show that Influpaint generates realistic, diverse epidemic trajectories and achieves forecast accuracy that is competitive with leading ensemble methods in retrospective evaluation. In real-time evaluation during the 2023–2025 U.S. CDC FluSight challenges, performance improved substantially across seasons, with highly accurate but somewhat overconfident projections in 2024–2025. The best performance was achieved with a training dataset containing 30% surveillance and 70% simulated trajectories. These results show that diffusion models can capture important spatiotemporal structure in influenza dynamics and provide a flexible framework for probabilistic infectious disease forecasting.

## 1 Introduction

Infectious disease forecasting can inform timely public health action, such as resource allocation and interventions. Producing reliable forecasts is challenging. Epidemics are dynamic, complex systems shaped by intrinsic randomness, changing population behaviors, environmental factors, and reporting delays. Accurate forecasting requires models that can handle non-stationary dynamics and limited data availability while capturing the full spectrum of plausible future trajectories.

Most forecasting approaches are based on mechanistic or statistical models. Mechanistic models incorporate epidemiological knowledge, but are prone to create unrealistic emergent dynamics and can be overly reliant on baseline assumptions. Statistical time-series methods excel at identifying historical patterns but can be brittle when underlying processes shift and are of limited utility when confronted with a novel pathogen. Both mechanistic and statistical approaches often under-represent the multimodal uncertainty seen in influenza seasons.

The new generation of generative AI models includes promising approaches for modeling high-dimensional distributions. In particular, denoising diffusion probabilistic models (DDPMs) have demonstrated paradigm-shifting performance in image and audio synthesis [6, 3]. Their ability to produce coherent samples conditioned on partial observations makes them well suited to spatiotemporal epidemic forecasting. While there has been limited work using Generative Adversarial Networks (GANs) and other deep learning methods for epidemic forecasting [17, 22, 8], the capacity of diffusion models to capture nuanced uncertainty and diverse epidemic dynamics remains unexplored.

Here we introduce **Influpaint**, a generative framework that applies DDPMs to influenza forecasting in the United States. Influpaint represents an influenza season as a two-dimensional image, with time on one axis, location on the other, and pixel intensity reflecting incidence. This representation enables the direct use of modern image-generation techniques, while conditioning on observed history is achieved through an inpainting procedure that enforces coherence between known observations and generated futures [24]. We describe the Influpaint architecture and training datasets, evaluate its ability to reproduce realistic epidemic dynamics, and assess both its prospective forecasting skill and its performance over the past three FluSight seasons (2022–2025). We benchmark alternative model formulations and quantify the contributions of key design choices such as dataset composition and inpainting schedule.

## 2 Results

### 2.1 Unconditional generation of realistic influenza seasons

**Figure 1:**
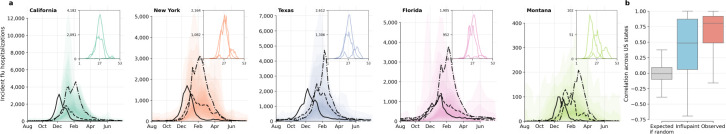
Influenza trajectories generated by Influpaint. **a.** Weekly hospital admissions are summarized by quantiles across 512 generated trajectories for California, New York, Texas, Florida, and Montana; three example trajectories are highlighted in the inset. Black lines show realized historical influenza seasons from NHSN data for each state. **b.** Pairwise cross-state weekly incidence correlations from Influpaint-generated trajectories, compared with a time-permuted null expectation and with observed correlations from recent historical seasons.

When generating unconditional trajectories (i.e., seasonal trajectories with no observed data), Influpaint produces novel (non-training) samples that respect known influenza epidemic patterns, including varying periods of increase and decline with clear seasonal peaks (Figure 1.a). This indicates that the model has learned a rich, high-dimensional representation of influenza dynamics. Visual examination of synthetic seasons suggests a high degree of realism and diversity. For instance, Influpaint generates seasons with a single high-intensity peak as well as seasons with bimodal epidemic curves, two patterns previously observed (e.g., in 2023–2024 and 2024–2025). This variety is difficult to replicate with classic epidemic models. The model further produces seasons with unusually early or late peaks, as well as seasons with low, geographically scattered incidence. The envelope produced by 512 trajectories contains the range of incidence seen in recently observed seasons (2022–2023, 2023–2024, and 2024–2025).

Likewise, Influpaint recovers much of the qualitative structure of multi-state epidemic timing (Figure 1b), with projections showing stronger spatial synchrony than expected by chance (mean inter-state correlation 0.455), though still below the levels observed in the 2023–2024 and 2024–2025 influenza seasons (mean inter-state correlation 0.816).

### 2.2 Forecasting via temporal inpainting

**Figure 2:**
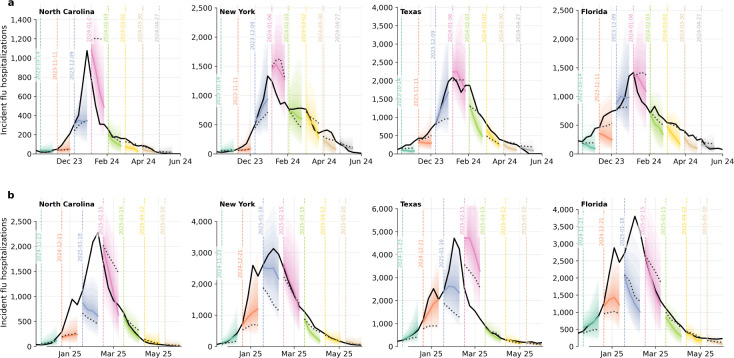
4-week-ahead forecasts. **a.** Each panel shows 4-week-ahead forecasts for North Carolina, New York, Texas, and Florida at multiple reference dates (colored dashed vertical lines). Forecast uncertainty is summarized by quantiles (colored fan) and the median (colored line) from 512 conditional trajectories. The solid black line shows the observed final values. For the same reference dates, the FluSight ensemble forecast is shown as a dotted line. **b.** Same as panel a, but for the 2024–2025 influenza season.

We next evaluate its core application: forecasting future trajectories conditioned on partially observed seasons. Figure 2 shows 4-week-ahead forecasts, a time horizon frequently used in infectious disease forecasting competitions, alongside the ensemble projections from FluSight, which are commonly regarded as among the best and most consistent available forecasts. Influpaint forecasts often anticipate turning points, with trajectories steepening and then drifting downward near seasonal maxima.

Quantitatively, Influpaint performed competitively with the CDC FluSight multimodel ensemble across the two prospective evaluation seasons (2023–2024 and 2024–2025). We evaluate probabilistic accuracy using the Weighted Interval Score (WIS), a proper scoring rule commonly used in influenza forecasting, for which lower values indicate better performance [2]. Under optimal conditions, Influpaint achieved a total WIS of 185,441 in 2023–2024 (ranked 5^th^ of 32) and 450,797 in 2024–2025 (ranked 8^th^ of 42), compared with 197,834 and 554,066 for the FluSight ensemble (ranked 8^th^ and 20^th^, respectively). Coverage was well calibrated, though slightly overconfident: empirical 50% and 90% interval coverages were 0.48 and 0.85 in 2023–2024 and 0.51 and 0.85 in 2024–2025, compared with 0.50 and 0.86 for the ensemble in 2023–2024 and 0.50 and 0.76 in 2024–2025. Although this comparison is imperfect because Influpaint forecasts were generated retrospectively using the latest available data rather than under real-time reporting constraints, it nonetheless shows that a diffusion-based generative model can achieve accuracy and calibration comparable to those of leading operational ensembles while offering coherent, sample-based representations of uncertainty and diverse epidemic trajectories.

**Figure 3:**
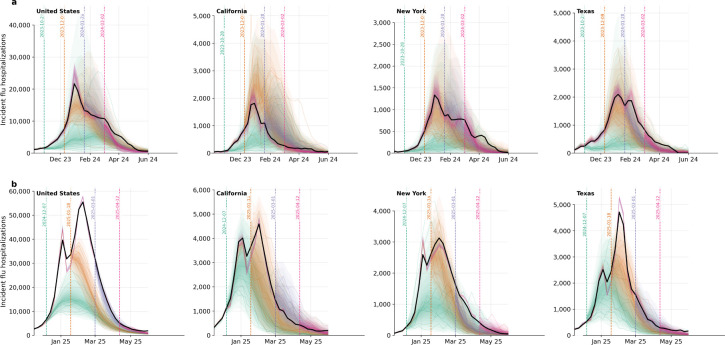
Full-season forecasts. **a.** Each panel for the United States, California, New York, and Texas shows reported hospitalizations (black line) and reference forecast dates (colored dashed vertical line), followed by forecast quantiles from 512 conditional trajectories (colored bands/lines) representing the probabilistic forecast. 10 example trajectories are highlighted as thin colored lines. **b.** Same as panel a, but for the 2024–2025 influenza season.

Figure 3 extends the forecast horizon to the end of the season. Early in the season, the predictive distribution is multimodal, reflecting divergent but plausible seasonal trajectories. As intended, uncertainty is tight near the beginning of the forecast and retains reasonable empirical coverage as the horizon grows. Qualitatively, Influpaint preserves informative seasonal signal at long horizons while adapting quickly as weekly observations reveal the dominant trajectory. For longer horizons, performance predictably degrades relative to short-term ensemble forecasts.

### 2.3 Training data mix and model formulation determine forecast skill

Having established prospective forecast performance, we next quantified which design choices were most responsible for Influpaint’s skill. We ran one-at-a-time ablations on 1–4-week-ahead state forecasts in 2023–2024 and 2024–2025 and evaluated each variant.

The composition of the training data had an important effect. We compared four training sets: surveillance only (CDC FluView and FluSurv), simulation only (Flu Scenario Modeling Hub and flepiMoP trajectories [11, 13]), and two hybrids (30%/70% and 70%/30% surveillance/simulation). Both hybrids outperformed single-source training, and the 30% surveillance / 70% simulation mix gave the best WIS profile across seasons and horizons (Table 1). Despite its larger size (1,240 unique training samples, compared with 20 for the surveillance-only dataset), the simulation-only dataset produced worse performance.

Other formulation choices had measurable effects. Notably, using 500 denoising steps rather than 200 and using square-root scaling both improved forecasting performance. Additional training perturbations intended to enrich the training dataset (Poisson resampling, temporal padding, intensity scaling) generally worsened performance. By contrast, alternative U-Net variants and inpainting schedules produced only small differences (Supplementary Figure 1).

### 2.4 Realized performance on the FluSight Challenge

To evaluate Influpaint in an operational setting, we submitted its forecasts under the team name UNC_IDD-Influpaint to the U.S. CDC FluSight forecasting challenge for the 2022–2023, 2023–2024, and 2024–2025 influenza seasons. FluSight is conducted in real time: each week, participating models submit probabilistic forecasts of influenza hospital admissions at the national and state levels, which are evaluated once observations become available. Across the three submitted seasons, Influpaint performance improved markedly as the algorithm matured, eventually performing among the top models in the most recent season (2024–2025).

Our first FluSight participation, for the 2022–2023 season, used an early Influpaint implementation based on the RePaint inpainting algorithm. This version exhibited boundary misalignment between observed and generated weeks, which degraded forecast calibration. The model ranked 9^th^ of 17 participating systems, with 50% prediction-interval coverage of 0.36 [15] (9^th^/17 on Maximum Absolute Error, MAE, and 10^th^/17 on relative WIS, where it is compared with a constant baseline).

Across the two most recent FluSight seasons, Influpaint showed marked improvement as the framework matured and the training data and conditioning algorithms were refined. Among models submitting at least 70% of weekly forecasts (excluding FluSight ensemble variants), UNC_IDD-InfluPaint ranked near the bottom of the leaderboard in 2023–2024 (Absolute WIS = 61.9, rank 24/27; Relative WIS = 1.10, rank 20/27; MAE = 87.8, rank 24/27), with empirical 50% and 90% coverages of 28.7% and 71.7%. In contrast, in 2024–2025 the model achieved substantial gains in sharpness and accuracy, ranking first of 36 models for both Absolute WIS (97.9) and MAE (128.3), and eleventh for Relative WIS (0.71). However, coverage was substantially below target (14.8% at 50%, 52.6% at 90%), indicating that while forecasts became sharper, uncertainty was underestimated.

### 2.5 Generalization to heterogeneous masking patterns

Because conditioning is implemented through inpainting, Influpaint can enforce arbitrary observation masks at sampling time: any subset of spatiotemporal entries (weeks × locations) can be fixed to observed values, and the remaining entries are sampled from the learned conditional distribution. This makes the approach well matched to temporal reporting gaps and partial spatial coverage.

**Figure 4:**
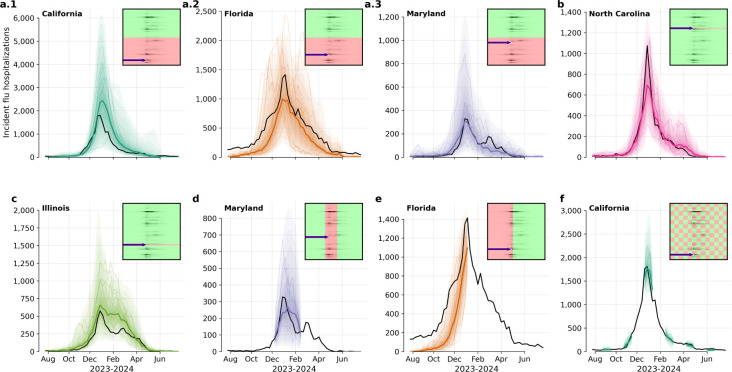
Robustness to mask design. Black curves show observed hospitalizations during the 2023–2024 season; colored fans and lines show Influpaint predictive quantiles and medians. Insets show the conditioning mask for each panel, where green values are observed and red values are hidden and reconstructed by Influpaint. The arrow in each inset indicates which state/row is shown in the graph. **a.1–3.** Half-subpopulation spatial mask for California (a.1), Florida (a.2), and Maryland (a.3). **b.** Leave-one-state-out mask for North Carolina. **c.** Leave-one-state-out mask for Illinois. **d.** Midseason gap mask for Maryland. e. Past-only conditioning mask for Florida. **f.** Checkerboard spatial-temporal mask for California.

Empirically, Influpaint handles partial information well and reconstructs coherent, plausible trajectories across diverse mask designs (Figure 4). For spatial reconstruction, a half-map mask withholding 50% of locations preserves broad peak-timing and peak-magnitude structure (Figure 4a.1–a.3), and leave-one-state-out masks infer plausible trajectories for fully unobserved states from surrounding context (Figure 4b,c). Temporal reconstruction is also informative: when only part of a season is observed, the model recovers plausible peak timing and intensity with wider uncertainty, reflecting the limited constraints (Figure 4d). As expected, reconstruction degrades when local dynamics deviate strongly from neighboring-state patterns, as illustrated for Florida in 2023–2024 under future-only conditioning (Figure 4e). We further illustrate this flexibility with a checkerboard spatiotemporal mask (4 weeks × 4 states; Figure 4f).

## 3 Discussion

This study shows how diffusion models can be used for infectious disease forecasting. By encoding each influenza season as a two-dimensional time-by-location frame, Influpaint learns a joint spatiotemporal distribution and can generate entire seasons that are realistic in both timing and spatial dynamics. From DDPMs it inherits a flexible inductive bias and the ability to span a diverse set of plausible outcomes, which is central to expressing uncertainty in forecasts. To our knowledge, this is the first application of DDPMs to infectious disease forecasting.

Influpaint achieved competitive performance in the CDC FluSight challenge, even in years perturbed by the COVID-19 pandemic, and performance improved substantially across three seasons as the framework matured. While our method did not outperform many existing approaches which often incorporate decades of epidemiological expertise, we show that a purely deep-learning generative model can produce competitive real-time forecasts. Moreover, across FluSight seasons, no single method consistently outperforms alternatives across all targets and epidemiological contexts. Achieving this level of performance provides a proof of concept for the potential of diffusion models. Further, there are clear pathways to improve performance, such as training on the forecast task more directly, and including nowcasting in the forecasting pipeline.

We identified dataset composition as a major determinant of forecast performance: models trained on hybrid datasets containing both surveillance and simulation data consistently outperformed single-source datasets, with the 30%/70% surveillance/simulation mix giving the strongest forecasting performance. In other fields, such as weather forecasting, synthetic datasets have been central to the development of deep learning methods [10, 16]. Here, Influpaint leverages the rich collection of influenza models assembled by the Flu Scenario Modeling Hub [13].

Finally, Influpaint can generate conditional samples under arbitrary temporal and spatial observation patterns. This flexibility is inherited from DDPM inpainting methods [19] and allows the same trained model to support a variety of public-health-relevant tasks, such as interpolating missing weeks and/or locations. Moreover, Influpaint produces coherent trajectory ensembles, so downstream quantities such as peak timing, peak magnitude, and cumulative burden are obtained directly from sampled predictive distributions. The same architecture can be deployed across geographic scales when compatible data are available, enabling a unified workflow from national to subnational forecasting.

Like other forecasting models, this study has two important limitations: it is difficult to assess the real-time performance of the final optimized algorithm across multiple seasons, and we cannot fully disentangle limitations specific to this implementation from limitations of the broader diffusion-based forecasting framework. In addition, several Influpaint-specific factors limit its widespread utility in its current form. Performance remains bounded by the diversity and realism of the training data. The hybrid dataset mitigates data scarcity but carries biases from both surveillance and modeling sources. Our ablations suggest that simulated trajectories are an important component for deep-learning forecasts, as in other domains. At the same time, simulation-only training underperformed surveillance-only training despite the larger number of samples, raising concerns about the quality and diversity of available synthetic data and their ability to capture the range of epidemic structures. Influpaint also does not yet handle reporting delays or backfill correction, which may affect real-time applications in which data are revised over time. Moreover, interpretability remains a challenge: as a deep generative model, Influpaint does not provide mechanistic explanations for its predictions. Finally, computational cost is substantial: training requires several hours on high-end GPUs, and producing full ensembles of trajectories is slower than for many other models.

Future extensions could improve forecasting performance, at the expense of flexibility, by training conditional diffusion models directly for forecasting rather than conditioning during generation. The current framework could also be expanded by including auxiliary channels such as climate, mobility, or virological data to inform dynamics through additional covariates. Another promising area is the combination of Influpaint with mechanistic models, either by using mechanistic outputs as additional covariates or by using diffusion models to learn residual processes around mechanistic forecasts. Beyond influenza, the Influpaint framework is general and could be extended to other pathogens with rich spatiotemporal data, including RSV, COVID-19, and dengue.

Influpaint demonstrates that recent developments in generative AI, and diffusion models in particular, can deliver competitive and flexible infectious disease forecasts. As surveillance and simulation data ecosystems continue to improve, generative diffusion models may become an important component of epidemic forecasting systems, providing a flexible method for anticipating and understanding disease dynamics.

## 4 Materials and Methods

### Overview

The architecture of Influpaint follows three steps: epidemic trajectories are first encoded into image-like representations, then used to train a generative model on a hybrid of surveillance and synthetic influenza data, and finally conditioned on past observations so the model can generate probabilistic forecasts.

### 4.1 Epidemic trajectories as images

**Figure 5:**
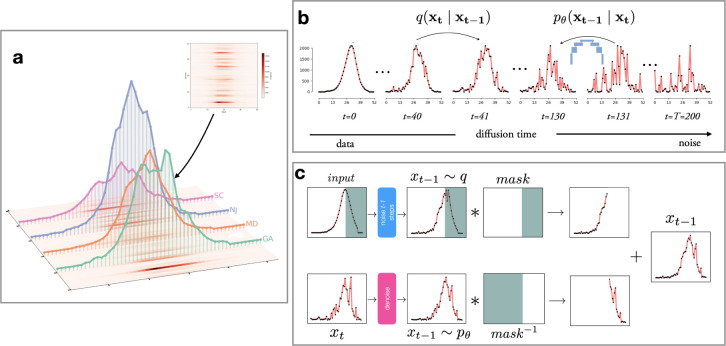
Methods behind Influpaint. **A.** Epidemic seasons are represented as images with time and space as axes, and pixel intensity corresponding to incident hospitalizations. **B.** Denoising Diffusion Probabilistic Models generate data by training a U-Net to restore an image that has been progressively corrupted by Gaussian noise. **C.** For forecasting, the ground truth and a mask (green: observed values to preserve) are provided. At each denoising step, we stitch the noised ground truth and the intermediate denoised sample to obtain the updated trajectory.

Influenza seasons are represented as two-dimensional arrays (or *frames*) X of shape 52×L, where the *x*-axis corresponds to time in weeks and the *y*-axis to locations (Figure 5.A). Each element $X_{ij}$ denotes a reported epidemiological quantity (e.g., incident influenza hospitalizations) in week i for location j. In the U.S. influenza context, L=51 represents all 50 states plus D.C.

This encoding is analogous to a grayscale image, with pixel intensity proportional to incidence, which enables the direct use of image-generation models. Although the temporal and spatial axes do not have the same semantics, and human mobility or spatial dependence may not be fully captured by convolution kernels, we find that diffusion-model architectures generalize effectively to epidemic generation.

### 4.2 Training data

Diffusion models require a large number of high-quality samples for training, but historical influenza datasets are limited, incomplete, and heterogeneous. To provide sufficient training data, we built a hybrid dataset combining reported hospitalization data and synthetic epidemic trajectories generated by mechanistic influenza models. It features:

- CDC FluView (via the Delphi Epidata API; percent influenza-like illness).
- CDC FluSurv-NET hospitalizations (via the Delphi Epidata API; extended to all states by population weighting);
- Synthetic influenza trajectories from multiple modeling teams, as archived in the Flu Scenario Modeling Hub:
  - Round 4 (2023–2024): 4 models, 600 trajectories each across 6 scenarios,
  - Round 5 (2024–2025): 7 models, at least 600 trajectories each across 6 scenarios
  - Round 1 (2022–2023): flepiMoP model, 1,200 trajectories across 4 scenarios,

#### Frame library.

To make all frames complete seasons (all locations and weeks), we assembled seasons from available sources while preserving cross-state relationships and realistic temporal structure. To avoid oversampling the synthetic dataset, we selected 20 trajectories per scenario per model. The final frame library contains 1,080 frames from SMH rounds 4 and 5 and 160 from flepiMoP round 1, in addition to the 20 surveillance frames (13 years of FluView and 7 years of FluSurv).

#### Dataset compositions.

From this frame library, we constructed four datasets with varying surveillance/modeling mixes:

- 100% surveillance (20 frames, repeated to a dataset of size 520, larger than our batch size),
- 100% modeling (1,240 frames),
- 30% surveillance / 70% modeling (1260 unique frames, repeated to 3,000),
- 70% surveillance / 30% modeling (1,260 unique frames, repeated to 3,000).

#### Transforms and augmentations.

To harmonize data sources, FluView percentages of influenza-like illness were rescaled using the distribution of SMH peak intensities. We applied square-root scaling before rescaling all frames to the [0,2] range. Inspired by image-generation augmentation methods [9], we explored targeted augmentations designed to enrich variability while preserving realism: Poisson resampling (observational noise), temporal padding (±4 or ±15 weeks), and intensity rescaling by a factor α with either narrow (0.7–1.3) or wide (0.1–1.9) ranges.

We therefore evaluated four dataset compositions, two transforms (linear vs square-root), and four enrichment schemes (none; Poisson only; Poisson + narrow pad/scale; Poisson + wide pad/scale).

As detailed in the SI, performance was best without augmentation on the 30% surveillance / 70% modeling dataset.

### 4.3 Generative model: denoising diffusion probabilistic model

A Denoising Diffusion Probabilistic Model (DDPM) consists of two processes. The forward (noising) process repeatedly adds Gaussian perturbations, progressively destroying the signal until only noise remains after *T* steps. The backward process applies a neural network trained to remove that noise step by step, so generation can start from pure noise and produce new, realistic seasons.

DDPMs were introduced for image generation by Ho et al. [6] and extended by Dhariwal and Nichol [3]. Below, we summarize the governing principles as used in Influpaint and refer the reader to these works for the full derivations.

#### Forward (noising) process.

We start by drawing a trajectory from our data distribution $x_{0}∼q\left(x_{0}\right)$ and repeatedly perturb it with Gaussian noise according to a variance schedule $\beta_{1},\ldots ,\beta_{T}$. The variance schedule is defined so that after all forward steps, when t=T, the trajectory is indistinguishable from white noise, as shown in Figure 5.B. After t steps of independent noising, the result can be expressed in closed form as a Gaussian:

(1) $$q\left(x_{t}|x_{0}\right)=N\left(x_{t};\sqrt{{\bar{\alpha }}_{t}}x_{0},\left(1-{\bar{\alpha }}_{t}\right)I\right),\;\text{where}\;{\bar{\alpha }}_{t}=\prod_{s=1}^{t}\left(1-\beta_{s}\right).$$

The mean is the original trajectory scaled by $\sqrt{{\bar{\alpha }}_{t}}$, and the variance term accumulates the injected noise.

#### Reverse (denoising) process.

Generation starts by drawing from a pure Gaussian prior, $p\left(x_{T}\right)=N\left(x_{T};0,I\right)$. A neural network $\epsilon_{\theta }\left(x_{t},t\right)$ is trained to predict the noise added at each step. By subtracting this estimate, we move one step toward a clean trajectory. This produces a learned Markov chain:

(2) $$p_{\theta }\left(x_{t-1}|x_{t}\right)=N\left(x_{t-1};\mu_{\theta }\left(x_{t},t\right),\sigma_{t}^{2}I\right),$$

where the mean subtracts the estimated noise at step t, and the variance is the posterior variance following the derivation in [6]:

(3) $$\mu_{\theta }\left(x_{t},t\right)=\frac{1}{\sqrt{1-\beta_{t}}}\left(x_{t}-\frac{\beta_{t}}{\sqrt{1-{\bar{\alpha }}_{t}}}\epsilon_{\theta }\left(x_{t},t\right)\right)\;\sigma_{t}^{2}={\tilde{\beta }}_{t}:=\frac{1-{\bar{\alpha }}_{t-1}}{1-{\bar{\alpha }}_{t}}\beta_{t}.$$

In the final generation step $\left(x_{1}\to x_{0}\right)$, the variance is set to zero.

#### Training objective.

We train our neural network $\epsilon_{\theta }\left(x_{t},t\right)$ by sampling a frame from the training dataset, applying the forward transform (Eq. 1) at a timestep t∼Unif{1,…,T}, and optimizing its ability to recover the exact noise realization. Drawing ϵ∼N(0,I), Ho et al. derive the following objective, which we minimize:

(4) $$L(\theta )=E_{x_{0},\epsilon ,t}\left[\epsilon -\epsilon_{\theta }{\left(x_{t},t\right)}_{2}^{2}\right]\;\text{where}\;x_{t}=\sqrt{{\bar{\alpha }}_{t}}x_{0}+\sqrt{1-{\bar{\alpha }}_{t}}\epsilon .$$

#### Network architecture.

We implement $\epsilon_{\theta }$ as a convolutional U-Net [18]: an encoder-decoder architecture that repeatedly halves spatial resolution while expanding channel depth, then mirrors the process with learned upsampling and skip connections so fine-scale details are combined with global context. Each block stacks ResNet convolutional residual layers [5], group normalization [23], and multi-head self-attention [21]. Sinusoidal timestep embeddings enter every block so the network always knows which diffusion step it is denoising. We trained both linear and cosine noise schedules with T∈{200,500} steps (we also tried 800, but these models did not converge within our time constraints), comparing four U-Net architectures: a compact 3-scale ResNet (1, 2, 4), a deeper 4-scale ResNet (1, 2, 2, 4), a ConvNeXt-inspired variant (1, 2, 2, 4; [12]), and a 5-scale ResNet (1, 2, 4, 4, 8). The numbers in parentheses represent the channel multipliers across resolutions. Every variant shares the same training objective.

### 4.4 Conditioning via Inpainting: CoPaint Algorithm

To produce forecasts, we use inpainting algorithms that modify the generation process so the final trajectory is conditioned on observed data. We first implemented RePaint [14], a DDPM sampler that repeatedly overwrites the observed region during reverse diffusion so the mask is always satisfied (see Figure 5.C). As in the generative process above, each iteration starts from the current sample $x_{t}$, but we replace the known pixels with their ground-truth values noised to the same time step t, and then denoise one step backward. While RePaint shows impressive coherence in image-generation tasks and is proven to generalize to unseen masks [19], Rout et al. uncovered an alignment bias that leaves a visible incoherence at the observed-forecast boundary, which affected Influpaint performance during the 2022–2023 FluSight season.

CoPaint [24] addresses this mismatch by introducing a Bayesian formulation that jointly updates both revealed and unrevealed pixels at every denoising step, ensuring global coherence and preserving the diffusion trajectory. We apply CoPaint using its optimized DDIM (O-DDIM) sampler, an extension of the Denoising Diffusion *Implicit* Model (DDIM) [20] that augments the deterministic reverse process with (i) a masked latent-refinement step at each time index and (ii) an optional time-travel schedule with re-noising that revisits earlier timesteps to reinforce the conditioning. These improvements lead to coherent inpainting and a smooth transition between masked and known regions. We refer the reader to the Supplementary Information for our investigation of the inpainting schedule.

### 4.5 Implementation Details

Influpaint was trained and evaluated on Nvidia L40 and A100 GPUs. With a batch size of 512, Influpaint requires at least 20 GB of GPU RAM. The iterative process used to inpaint a single forecast, for any mask, takes between 20 and 40 minutes to generate an ensemble of 512 conditioned trajectories. The framework is developed in Python using PyTorch, with implementations of CoPaint, RePaint, DDIM, and DDPM derived from their respective publications.

We relied on the hubverse [7] to interact with other model forecasts, the scoringutils R package [1] to score models, and the Delphi Epidata API [4] to fetch FluView data for construction of the Influpaint dataset.

### 4.6 Statistical analysis

For all forecasts submitted to FluSight or used in this work, Influpaint generates a predictive distribution represented by an ensemble of n=512 sampled trajectories. We converted these samples to the standard FluSight quantile format using 23 quantile levels: 0.01, 0.025, 0.05, 0.10, ..., 0.90, 0.95, 0.975, and 0.99. The same quantile grid was used throughout model evaluation. Forecast scores were computed in R with scoringutils, using the Weighted Interval Score (WIS), with lower values indicating better forecasts. Empirical interval coverage was also obtained from scoringutils; in the evaluation pipeline used for this study, the reported coverage metrics correspond to the 50% and 90% central prediction intervals. Coverage summaries reported in the manuscript are means of these per-forecast coverage indicators over the relevant set of forecast-observation pairs. For unconditional generation analyses, quantiles and envelopes were computed across n=512 generated trajectories. Pairwise cross-state weekly incidence correlations were calculated from state-level weekly hospitalization trajectories and summarized by their mean across state pairs.

## Supplementary Material

Supplement 1

Supplementary Information

Supplementary Information is available for this paper.

## Figures and Tables

**Table 1:** Effect of training-data composition on forecast skill. Each row is the Combined-season WIS for the single model obtained by changing only the dataset composition and keeping all other settings at their baseline values. Improvements are reported relative to the baseline 30% surveillance / 70% modeling mix, matching the dataset-composition entries in Supplementary Figure 1. Positive values indicate better performance than the baseline; negative values indicate worse performance.

Dataset composition	$N_{samples}$	Total WIS	Relative performance (%)
30% surveillance / 70% modeling	1,260	647,816	baseline
70% surveillance / 30% modeling	1,260	704,939	−8.82
100% surveillance	20	707,704	−9.24
100% modeling	1,240	724,125	−11.78

## Data Availability

The data used to train, run, and evaluate Influpaint are available on GitHub: https://github.com/ACCIDDA/Influpaint. Influpaint submissions are available in the CDC repositories https://github.com/cdcepi/Flusight-forecast-data for the 2022–2023 season and https://github.com/cdcepi/FluSight-forecast-hub for the 2023–2024 and 2024–2025 seasons, with model name UNC_IDD-InfluPaint.

## Code availability

The code used to train, run, and evaluate Influpaint is available on GitHub: https://github.com/ACCIDDA/Influpaint (DOI for this submission version will be given before publication). The pre-trained weights for the model analyzed in this publication are provided on Zenodo (DOI for this submission version will be given before publication), enabling direct use of Influpaint without retraining. Guidance for training and for applying the framework to data sources other than U.S. influenza is provided in the package documentation at https://accidda.github.io/Influpaint/.
