# Supplementary material for: Generative diffusion models for spatiotemporal influenza forecasting
Source: ArXiv. 2026 Apr 27:arXiv:2604.24913v1. Preprint. [Version 1] (PMC13142539)
Supplement: Supplement 1 [file NIHPP2604.24913v1-supplement-1.pdf]

A Supplementary Materials

A.1 Evaluation of competing model formulation

To identify the factors that most influence Influpaint's forecasting performance, we conducted a series of one-at-a-time ablations around a reference configuration. The baseline used a 500-step cosine DDPM with a three-scale U-Net, the 30% surveillance / 70% simulated data mix, square-root scaling, no training-time enrichment, and the short-jump CoPaint schedule with time travel. Forecasts were evaluated on 1–4-week horizons during the 2023–2024 and 2024–2025 FluSight seasons using the Weighted Interval Score (WIS).

Ablations varied diffusion depth and schedule, data composition, observation transforms, training enrichments, and inpainting schedules. We evaluated forecasting performance as the percent change in absolute WIS relative to the baseline (Figure 6).

The number of diffusion steps had the largest effect: models trained with T=500 diffusion steps outperformed shorter (T=200) schedules. Observation transform followed, with square-root scaling yielding better performance than linear scaling. Training-time enrichment (Poisson resampling, temporal padding, or intensity scaling) degraded performance, suggesting that such perturbations disrupt the inductive bias learned from the training data. In contrast, architectural variants within the same capacity class and alternative CoPaint schedules had minimal impact (Figure 6).

Inpainting schedules evaluated. We evaluate three CoPaint/ODDIM sampling schedules that differ only in how strongly and how often the observed data are enforced along the reverse diffusion trajectory: (i) a short jump (J=5) with time travel and five latent-refinement steps per time index (`celebahq_try3`); (ii) the same jump length without time travel and with two latent-refinement steps (`celebahq_noTTJ5`); and (iii) a longer jump (J=10) with time travel and two latent-refinement steps (`celebahq`). Here, *jump length* J controls the revisit frequency: after every J denoising steps the sampler revisits earlier diffusion times to reapply the conditioning so the mask remains satisfied. *Time travel* denotes a one-step forward re-noising between denoising updates, which empirically helps reconcile the observed (masked-in) history with the sampled future and reduces seams at the boundary. *Latent-refinement steps* are small gradient updates at each time index that nudge the current latent toward matching the observed region before proceeding. Because conditioning is performed entirely at prediction time (the diffusion model is trained unconditionally), any surveillance/forecast mask can be used without retraining; this is consistent with theoretical results on resampling-based diffusion inpainting generalization [19].

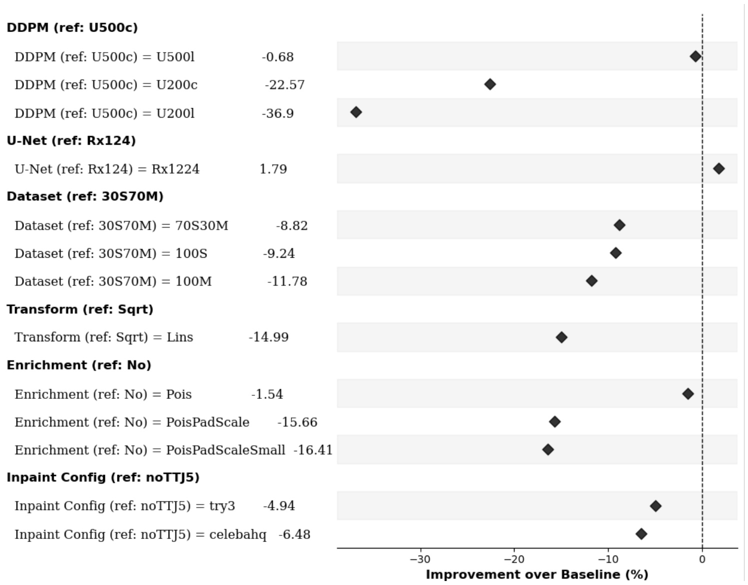

Figure 6: **Ablation effects on WIS.** Each point shows the mean paired change in absolute WIS for a model variant relative to the baseline configuration. Positive values indicate improved forecasting performance relative to the baseline, whereas negative values indicate worse performance. The first group reports diffusion-model denoising schedules, comparing 500 or 200 diffusion steps with either a linear (l) or cosine (c) variance schedule. The second group compares U-Net architectures, with *124* denoting a three-scale architecture with channel multipliers $(1, 2, 4)$ and *1224* a deeper four-scale architecture with channel multipliers $(1, 2, 2, 4)$. The dataset group compares training sets denoted *xxSyyM*, where *xx*% of samples come from surveillance data and *yy*% from modeled trajectories. The transform group compares square-root scaling of incidence values (*Sqrt*) with linear scaling (*Lins*). The enrichment group evaluates additional training perturbations: *No* (none), *Pois* (Poisson resampling), *PoisPadScaleSmall* (Poisson resampling with narrow temporal padding and intensity scaling), and *PoisPadScale* (Poisson resampling with wide temporal padding and intensity scaling). The final group compares the three inpainting configurations described in the main text.

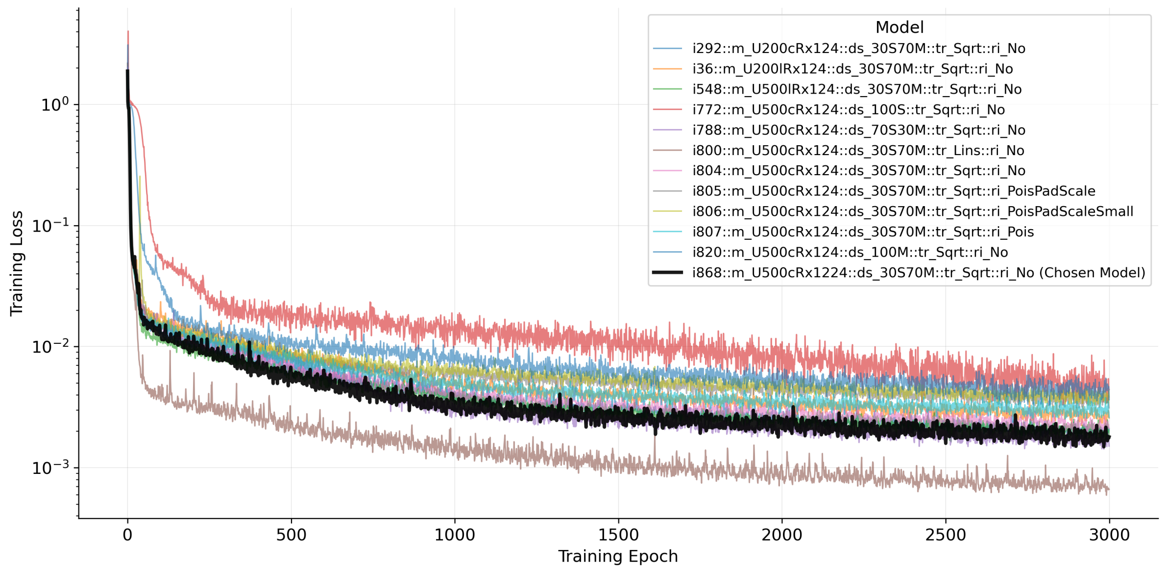

Figure 7: **Training losses during Influpaint calibration.** Curves show the training loss over 3,000 epochs for the candidate model configurations evaluated during calibration. The model selected for downstream analyses, based on its combined ranking in relative and absolute WIS, is highlighted in black.

A.2 Relationship between training loss and forecasting performance

During training, Influpaint's loss (Figure 7) measures performance on unconditional generation, not on forecasting directly. When we compared this loss with forecasting skill, we found a positive correlation, though not a strict ordering across models (Figure 8). This suggests that generative fit is informative about forecast quality, but does not fully determine it. Directly training diffusion models for forecasting may therefore improve performance further. Even so, these results show that training for unconditional generation already yields models with substantial forecasting skill.

A.3 Realized FluSight forecasts

We show a sample of real-time Influpaint forecasts submitted to the FluSight hub in Figure 9.

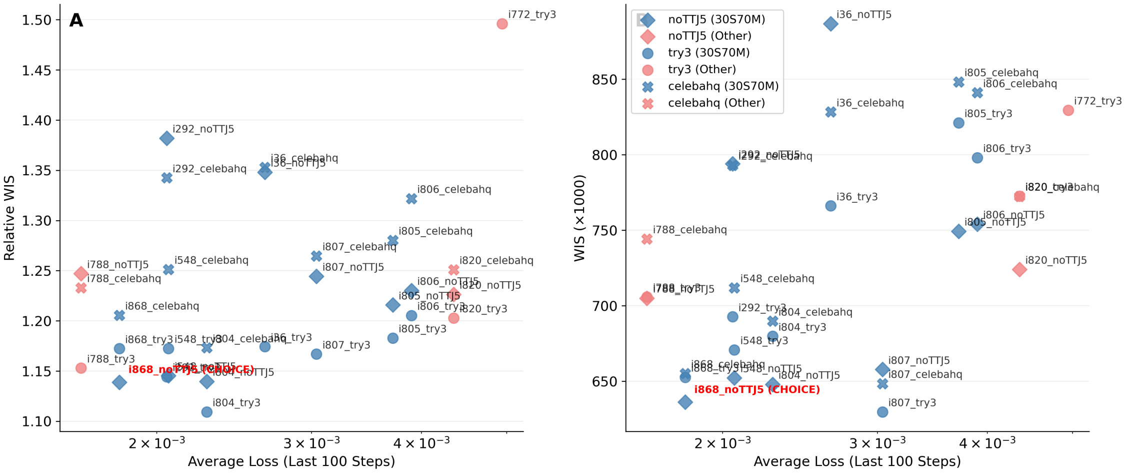

Figure 8: **Relationship between training loss and forecasting performance.** Final training loss is compared with relative WIS (**a**) and absolute WIS (**b**) across candidate model configurations. Each point represents one model variant, and the configuration selected for downstream analyses is highlighted in red.

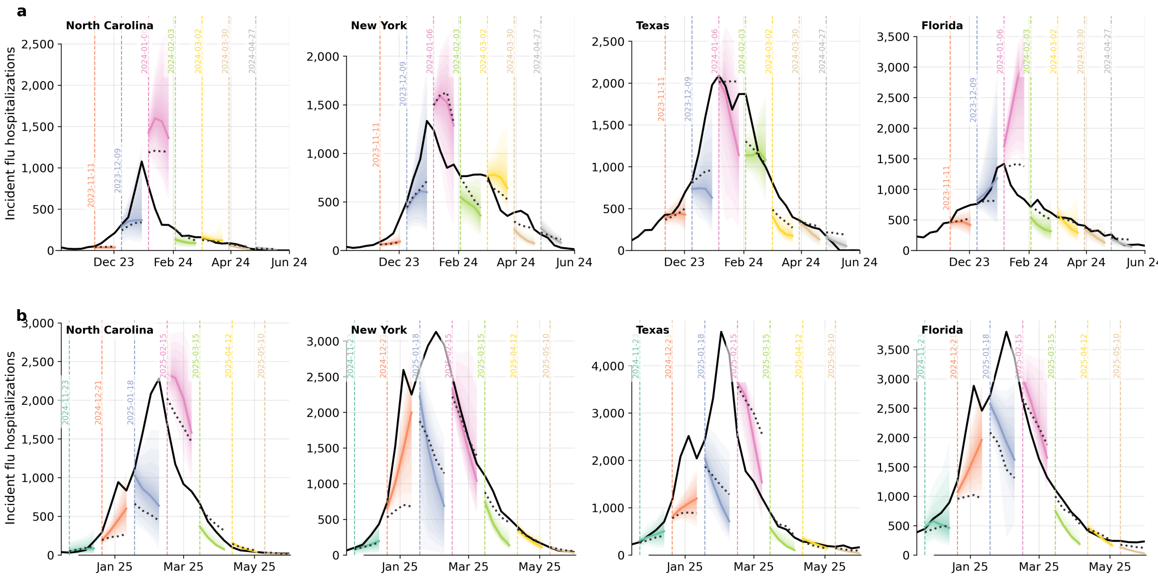

Figure 9: **a.** Each panel shows submitted 4-week-ahead FluSight forecasts from `UNC_IDD-InfluPaint` for North Carolina, New York, Texas, and Florida at the same reference dates used in the reference figure (colored dashed vertical lines). Forecast uncertainty is summarized by the submitted quantiles (colored fan) and median (colored line). The solid black line shows the observed final values. For the same reference dates, the FluSight ensemble forecast is shown as a dotted line. **b.** Same as panel a, but for the 2024–2025 influenza season.
